# Using Procedure Based on Item Response Theory to Evaluate Classification Consistency Indices in the Practice of Large-Scale Assessment

**DOI:** 10.3389/fpsyg.2017.01676

**Published:** 2017-09-26

**Authors:** Shanshan Zhang, Jiaxuan Du, Ping Chen, Tao Xin, Fu Chen

**Affiliations:** ^1^Faculty of Psychology, Beijing Normal University, Beijing, China; ^2^Collaborative Innovation Center of Assessment toward Basic Education Quality, Beijing Normal University, Beijing, China

**Keywords:** classification consistency index, item response theory, ability distribution, cut score, score transformation rule

## Abstract

In spite of the growing interest in the methods of evaluating the classification consistency (CC) indices, only few researches are available in the field of applying these methods in the practice of large-scale educational assessment. In addition, only few studies considered the influence of practical factors, for example, the examinee ability distribution, the cut score location and the score scale, on the performance of CC indices. Using the newly developed Lee's procedure based on the item response theory (IRT), the main purpose of this study is to investigate the performance of CC indices when practical factors are taken into consideration. A simulation study and an empirical study were conducted under comprehensive conditions. Results suggested that with negatively skewed distribution, the CC indices were larger than with other distributions. Interactions occurred among ability distribution, cut score location, and score scale. Consequently, Lee's IRT procedure is reliable to be used in the field of large-scale educational assessment, and when reporting the indices, it should be treated with caution as testing conditions may vary a lot.

## Introduction

For many large-scale assessments, examinees are generally classified into different categories by setting cut scores. Researchers use classification consistency as an important index to evaluate classification decisions (Brennan, 2004; Rudner, 2005; Yoo et al., 2009). Classification consistency (CC) index describes the degree to which examinees would be classified into the same performance categories over parallel replications of the same assessment (Lee, 2010), and it can be regarded as a reliability index of classification decisions.

Classification consistency makes it easy and convenient to describe the performance of examinees possessing different proficiency levels. For instance, with one cut score, examinees are typically classified based on a mastery/non-mastery decision, whereas with multiple cut scores, classification categories may have several choices such as basic, proficient, and advanced levels (Ercikan and Julian, 2002). In the practice of educational assessment, it is required to report the performance of examinees who would be classified in the same way on two applications of the procedure (American Education Research Association, 2014). The National Assessment of Educational Progress (NAEP) program followed the requirement and used the percentage of exact agreement as an index of the degree of decision consistency.

In order to illustrate how the CC indices work, a pass/fail test is used as an example. Obviously, the examinees taking this test will be classified into two groups: the “pass” group and the “fail” group. We assume that the probability of examinees passing both this test and its parallel test is p_11_ and the probability of examinees failing both this test and its parallel test is p_22_. Then the CC indices p can be obtained by: p = p_11_+p_22_. Hence, according to this formula, the CC indices reflect the proportion of consistent classification to examinees between the test and its parallel tests. However, due to the probability that examinees can be classified into the same category by chance (Cohen, 1960), the κ index was developed as the probability of a classification consistency excluding the influence of chance. The κ index is obtained by κ = (p−p_*c*_)/(1−p_*c*_), where p_*c*_ is the chance probability.

There are many methods for computing the CC indices (e.g., Livingston and Lewis, 1995; Brennan, 2004; Rudner, 2005; Lee, 2010). Basically, the common core of these methods is to generate a hypothesized parallel test of the targeted test for computing the CC indices. However, these methods use different models and estimation methods to obtain the CC indices. Generally, these methods can be classified into two groups: the classical test theory (CTT) methods and the item response theory (IRT) methods. The CTT methods are typically required to estimate the true score distribution based on the observed score distribution. Then the joint observed score distribution of two parallel tests generated by the true score distribution can be obtained. The joint observed score distribution can be used to estimate the CC indices. The CTT methods based on the true score distribution can be seen in Huynh (1976), Hanson and Brennan (1990), and Livingston and Lewis (1995). However, some procedures only considered each individual's probability of consistent classification to obtain the CC indices without estimating the true score distribution (e.g., Subkoviak, 1976; Brennan, 2004).

Although the methods under the CTT framework have been widely used, the IRT methods show a lot of advantages in estimating the CC indices (Lee et al., 2002; Yoo et al., 2009; Lee, 2010). Researchers found that the CC indices based on IRT usually tend to provide better fits and show larger estimated consistency than based on CTT (Lee et al., 2000, 2002). IRT has been widely used in test development, calibration, equating, and standard setting. Thus, it should be considered to analyze tests based on IRT by using non-CTT procedure to calculate the CC indices. Recently, the IRT-based methods have been quickly developed, such as Rudner method (Rudner, 2005), H-H method (Li, 2006), and Lee method (Lee, 2010). Rudner method expects that for any given true score, the corresponding observed score is normally distributed. H-H method uses the estimated item parameters and ability estimates to generate each examinee's test scores of two parallel tests. However, Rudner method and H-H method were developed based on dichotomous items. The newly developed Lee method (Lee, 2010) can be considered as a general framework to estimate the CC indices by IRT models for data comprising dichotomous and polytomous items. It makes no assumptions for the distribution of test scores and can be easily implemented. Based on this procedure, researchers proposed corresponding methods to estimate CC indices in multidimensional IRT (Yao, 2013) and cognitive diagnostic assessment (Cui and Gierl, 2012). For reporting CC indices in educational assessments, examples of using Lee's IRT procedure are also abundant (Wheadon and Stockford, 2010; Hendrawan and Wibowo, 2011). When comparing the quality of different measurement scales of internet addiction, Zhang and Xin (2013) used Lee's IRT procedure to obtain the CC indices. In addition, Lathrop and Cheng (2014) recently developed a new approach to estimate CC index non-parametrically by replacing the role of the parametric IRT model with a modified version of Ramsay's kernel-smoothed item response functions. However, their results showed that the non-parametric CC index performs similarly to Lee's procedure especially when the ability distributions are non-normal. Hence, we mainly focused on Lee method in this study.

Although Lee's procedure has been introduced in the practice of educational assessment, many factors can influence the performance of CC indices. However, only a few researches explored how practical conditions can affect the CC indices and took these comprehensive factors into consideration when reporting the indices.

The ability distributions provide the information of tests and easily influence the psychometric properties. Previous simulation studies have investigated the performance of CC indices for data simulated from IRT models with examinees who truly have normally distributed ability (Li, 2006; Wan et al., 2007; Adam and Hao, 2012). However, situations in practice are not always fit for the normal distribution. In some criterion-reference tests, the majority of candidates have already well mastered the knowledge skills measured by the assessment. In these cases, the ability distributions are always negatively skewed. The existing research achievements for the normal distribution are limited to be extended to other ability distributions.

Raw scores are very difficult to interpret, as they lack a reference scheme to interpret the performance of the examinees (Kolen and Lee, 2011). In order to obtain better interpretation and implementation of the assessment results, the scores are usually transformed by the linear or non-linear transformations from raw scores to scale scores (Kolen et al., 1992; Tong and Kolen, 2010). Data from various score scales result in various values of the standard errors of measurement and test reliability (Wang et al., 2000; Almehrizi, 2013). The previous studies have found that higher test reliability was related to higher values of CC indices (Huynh, 1976; Deng, 2011). It can be inferred that with various score scales, the CC indices may result in various performances.

In order to make consistent decisions and score reporting under comprehensive test conditions, the influence of these factors must be investigated. Due to the fact that students near the cut score location have larger misclassification errors (Ercikan and Julian, 2002), an interaction between the cut score location and ability distribution can be expected. Although Lee's procedure has been evaluated in simulation studies with normally distributed ability, the procedure is new and calls for further investigation. Given the fact that the true values of the indices and the true score distribution are unknown in real data, both simulation and empirical studies are necessary.

In this paper, a simulation study was conducted to evaluate the performance of CC indices with three ability distributions, two score scales, two cut score numbers, and two cut score locations. Two examples based on the real data from a large-scale assessment were used to evaluate the CC indices in real settings. Two purposes exist in this study. The first purpose is to verify the use of Lee's IRT procedure in a single administration of large-scale assessment, and the second purpose is to explore the factors affecting the CC indices.

## Methods

Based on the previous IRT methods developed by other researchers, Lee (2010) generalized their work and developed a general procedure. Using θ and *g*(θ) to denote the measured latent ability and its distribution, the marginal probability of the total summed score *X* can be obtained by:

(1) $$P(X=x)=\int_{-\infty }^{\infty }P(X=x|\theta )g(\theta )d\theta ,$$

where *P*(*X* = *x*|θ) is the conditional measurement error distribution, also called the conditional summed-score distribution, which indicates the probability that a summed score can be expressed by the multiplication of probabilities for item responses given θ .

Second, assume that the test score is obtained by summing all of the item scores through a particular way. In addition, the cut scores to classify the examinees into *k* mutually exclusive categories are defined as *x*_1_, *x*_2_, …, *x*_*k*−1_. Hence, a score smaller than *x*_1_ will be in the first category; a score larger than or equal to *x*_1_ and smaller than *x*_2_ will be in the second category, and so on. The conditional category probability can be computed by summing the conditional summed-score probabilities for all *x* values that belong to the category *h* as follows:

(2) $$p_{\theta }(h)=\sum_{x=x_{h-1}}^{x_{h}-1}P(X=x|\theta ).$$

Having the conditional category probability, we can know the probability of an examinee with a given ability value being classified into the same category on two parallel forms of a test. This is the conditional classification consistency index Φ_θ_ that can be computed as:

(3) $$\Phi_{\theta }=\sum_{h\;=\;1}^{\text{k}}{\left[p_{\theta }(h)\right]}^{2}.$$

Finally, having the conditional classification consistency index, we can then compute the classification consistency across all ability levels with the distribution of ability, *g*(θ). The marginal classification consistency index Φ is given by:

(4) $$\Phi =\int_{-\infty }^{\infty }\Phi_{\theta }g(\theta )d\theta .$$

As we mentioned earlier, there exists the probability that examinees can be classified into the same category by chance. Therefore, Φ_*c*_ is developed as the chance probability:

(5) $$\Phi_{c}=\sum_{h\;=\;1}^{K}{\left[p(h)\right]}^{2}.$$

Then *K* index is defined as the probability of a classification consistency that excludes the influence of chance:

(6) $$K=\frac{\Phi -\Phi_{c}}{1-\Phi_{c}}.$$

Both Φ and *K* are used as the CC indices in this study.

## Simulation study

### Data generation

A simulation study was conducted to evaluate the performance of CC indices based on Lee's procedure in which several different factors were manipulated. Data were simulated based on three ability distributions, two score scales, and a set of cut scores. The two-parameter logistic (2PL) IRT model (Birnbaum, 1968) and the graded response model (GRM) (Samejima, 1969) were employed to generate the unidimensional item response matrix. There were 20 dichotomous items scored 0 and 1, and 4 polytomous items with the score point of 0, 1, 2, 3, 4, and 5. The total summed raw score value was 40. The discrimination parameter *a* was drawn from ln *a* ~ *N*(0, 1) and *a*∈[0, 2.5], and the difficulty parameter *b* was drawn from *b* ~ *N*(0, 1) and *b*∈[−3, 3]. The number of examinees was fixed at 2,000. With each ability distribution, 100 replications were simulated. Thus, the simulation study generated a total of 300 metrics.

### Examinee ability distributions

This study investigated three different ability distributions. The first group of examinees' ability was drawn from a normal distribution with a mean of 0 and a standard deviation of 1. The examinee ability values of the positively skewed distribution were randomly drawn from the skewed distribution with the mean of 0, standard deviation of 1, kurtosis of 0, and skewness of 0.8. The examinee ability values of the negatively skewed distribution were randomly drawn from the skewed distribution with the mean of 0, standard deviation of 1, kurtosis of 0, and skewness of −0.8. The positively skewed distribution group had the mass of the distribution concentrated on the left of the ability scale and was designed to represent a group with slightly lower ability. On the contrary, the negatively skewed distribution group had the mass of the distribution concentrated on the right of the ability scale and was devised to represent a group with slightly higher ability. MATLAB 2012b was used to generate examinee ability values.

### Score scales

**Figure 1:**
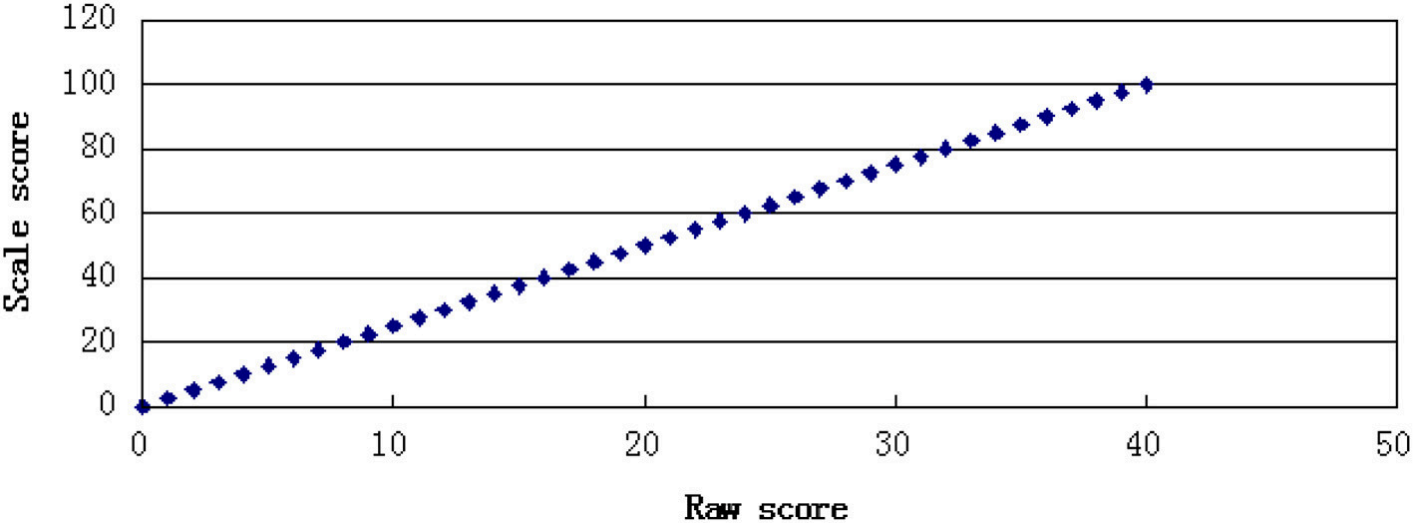
One to one transformation.

**Figure 2:**
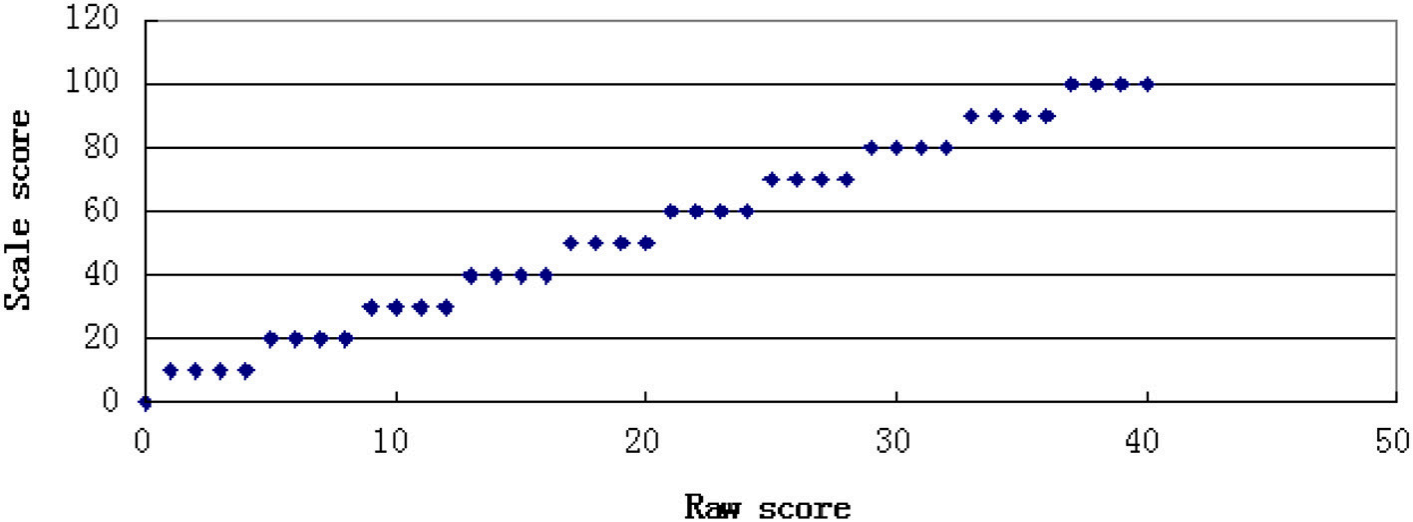
Many to one transformation.

In this study, two kinds of transformation rules were used to generate score scales. One was a linear transformation function in which the slope and distribution of the scale score were equal to the summed raw score. The other was a non-linear transformation function that resulted in an alteration of the distributional shape from the summed raw score values. The linear transformation function was a one-to-one transformation with every distinct summed raw score converted to a unique value of scale score, whereas the non-linear transformation function was a many-to-one transformation, in which several summed raw score values were converted to a unique value of scale score. The scale score had a total score of 100. The transformation rules are shown in Figures 1, 2.

### Cut-scores

The cut scores were fixed at the scale score metric with the values of 60 and 80. In the practice of Chinese achievement tests with a total score of 100, 60 and 80 are commonly used as the cut score values. By the test characteristic curve, the corresponded cut scores at θ metric were evaluated. Two cut score values were simultaneously applied to compute the CC indices. And the result of only applying one cut score would also be reported to see the effect of cut score numbers and locations. It was expected that CC indices would increase as the number of cut score decreased and that there may be interactions between the cut score locations and the ability distributions.

### Evaluation procedures

MATLAB 2012b was used to generate original item response matrices. Item parameters were calibrated using PARSCALE (Muraki and Bock, 1997) with the number of quadrature points set to 60 and the convergence criterion set to 0.005. The CC indices were calculated by the computer program IRT-CLASS (Lee and Kolen, 2008). In addition, EXCEL VBA and SPSS 20.0 were also used for the computations.

### Results

#### Marginal CC indices

Table 1 presents the estimates of the marginal CC indices for the various conditions. Situation 1, 2, and 3 were representations of the normal, positively skewed, and negatively skewed ability distribution, respectively. A and B indicate the two different cut scores. Using test characteristic curves and score transformation rules, the original cut scores at scale score metric were transformed to the metric of θ. The cut score values on the original scale of 100 and two transformed theta scales were shown in Table 2. A and B correspond to 60 and 80 on the score scale of 100, respectively. A/B means A and B were both used as the cut scores in the test. A or B means only cut score A or B was used in the test. The results for the marginal CC index, Φ, are shown on the left of the Table, whereas the results for the CC index excluding the influence of chance, *K*, are on right. The values in the brackets display the standard deviations of the 100 replications.

**Table 1:** Marginal classification consistency indices of simulation study.

		Φ **indices**			***K*** **indices**		
		**A/B**	**A**	**B**	**A/B**	**A**	**B**
Situation 1 [θ ~*N*(0,1)]	One to one	0.670 (0.004)	0.827 (0.003)	0.827 (0.004)	0.502 (0.006)	0.632 (0.006)	0.583 (0.007)
	Many to one	0.682 (0.004)	0.853 (0.003)	0.812 (0.004)	0.513 (0.006)	0.631 (0.007)	0.614 (0.006)
Situation 2 (Sk = 0.8)	One to one	0.728 (0.017)	0.840 (0.011)	0.879 (0.012)	0.578 (0.026)	0.670 (0.037)	0.707 (0.018)
	Many to one	0.712 (0.016)	0.833 (0.015)	0.866 (0.011)	0.561 (0.028)	0.617 (0.045)	0.717 (0.024)
Situation 3 (Sk = 0.8)	One to one	0.737 (0.013)	0.886 (0.006)	0.839 (0.008)	0.592 (0.019)	0.727 (0.017)	0.675 (0.016)
	Many to one	0.772 (0.011)	0.905 (0.004)	0.856 (0.008)	0.612 (0.019)	0.729 (0.016)	0.708 (0.017)

**Table 2:** Cut scores at different metric.

	**Cut score values at scale score**		**Corresponded** θ **(one to one)**		**Corresponded** θ **(many to one)**	
	**A**	**B**	**A**	**B**	**A**	**B**
Situation 1	60	80	−0.22	0.72	−0.42	0.47
Situation 2	60	80	−0.10	0.65	−0.33	0.51
Situation 3	60	80	−0.45	0.25	−0.61	0.06

Several important findings can be observed from Table 1. First, the performance of the two CC indices was different. Generally, *K* index was smaller than Φ. This means that compared to *K* index, Φ index leads to higher classification consistency level. This is due to that *K* index excludes the influence of chance probability. In addition, the standard deviations of *K* index were larger than that of Φ index.

Second, the performance of the CC indices was influenced by the number of cut scores. Compared to the condition that only one cut was used, when A and B were both used as the cut scores, the CC indices were smaller. This finding was consistent for all ability distributions and transformation rules. Therefore, it indicates that using too many cut scores in tests leads to lower classification consistency level for examinees.

Third, the performance of CC indices could be also influenced by different ability distributions. When both A and B were used as the cut scores, the CC indices tended to be smaller with the normal distribution than with the skewed distributions. This indicates that when two cut scores are used, the skewed student ability distributions lead to higher classification consistency level. In addition, generally, the negatively skewed distribution contributes to larger marginal CC indices compared to the normal and positively skewed distributions. Moreover, the standard deviations with the positively skewed distribution were larger than with the other two distributions, thus showing the unstable estimates of the values.

**Figure 3:**
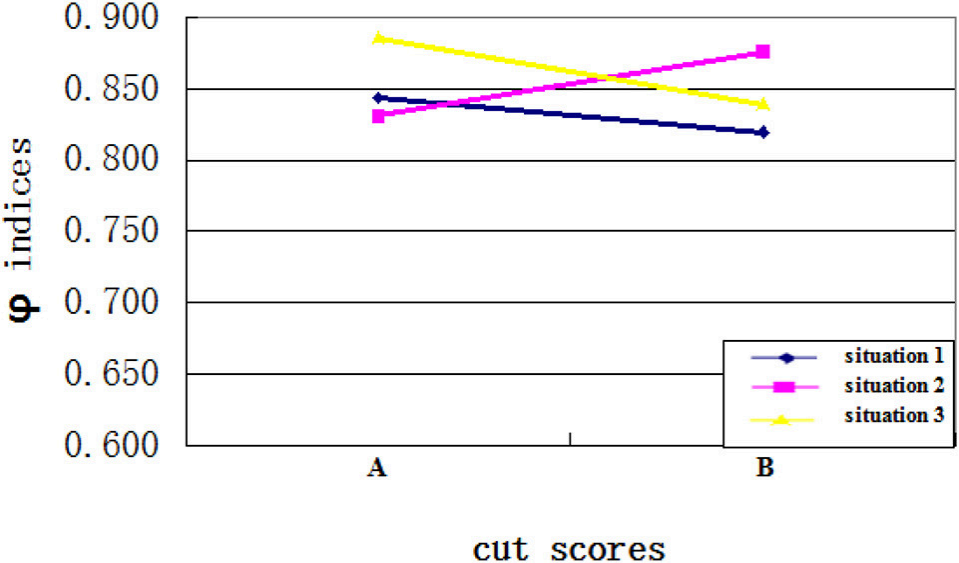
The interaction between cut score location and ability distribution for Φ indices.

**Figure 4:**
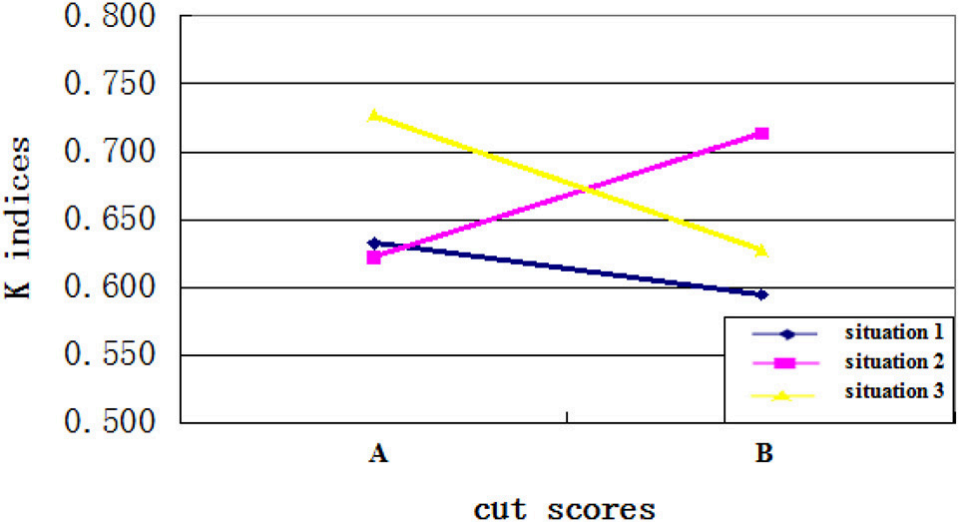
The interaction between cut score location and ability distribution for *K* indices.

Fourth, as expected, an interaction existed between the cut scores and the ability distributions. Specifically, when the cut score location on the ability scale and the placement of most test takers' ability were disparate, the CC indices would be larger. When the cut score location on the ability scale was close to the ability of most test takers, more misclassifications happened and the CC indices would be smaller. Figures 3, 4 clearly showed the interaction between the ability distributions and the cut score locations. For the negatively skewed distribution, the CC indices were larger with location A than with location B. As for the normal distribution, the CC indices were similar with locations A and B. For the positively skewed distribution, the CC indices were reversed with location B having higher values than location A. Both Φ and *K* indices showed the same trend of performance across these conditions. This finding suggests that when the ability distribution is positively skewed, a cut score with larger value leads to higher classification consistency level for the examinees, and vice versa. In other words, if most examinees have ability values close to the cut scores on the ability scale, then the classification consistency level will be lower.

#### Conditional CC indices

**Figure 5:**
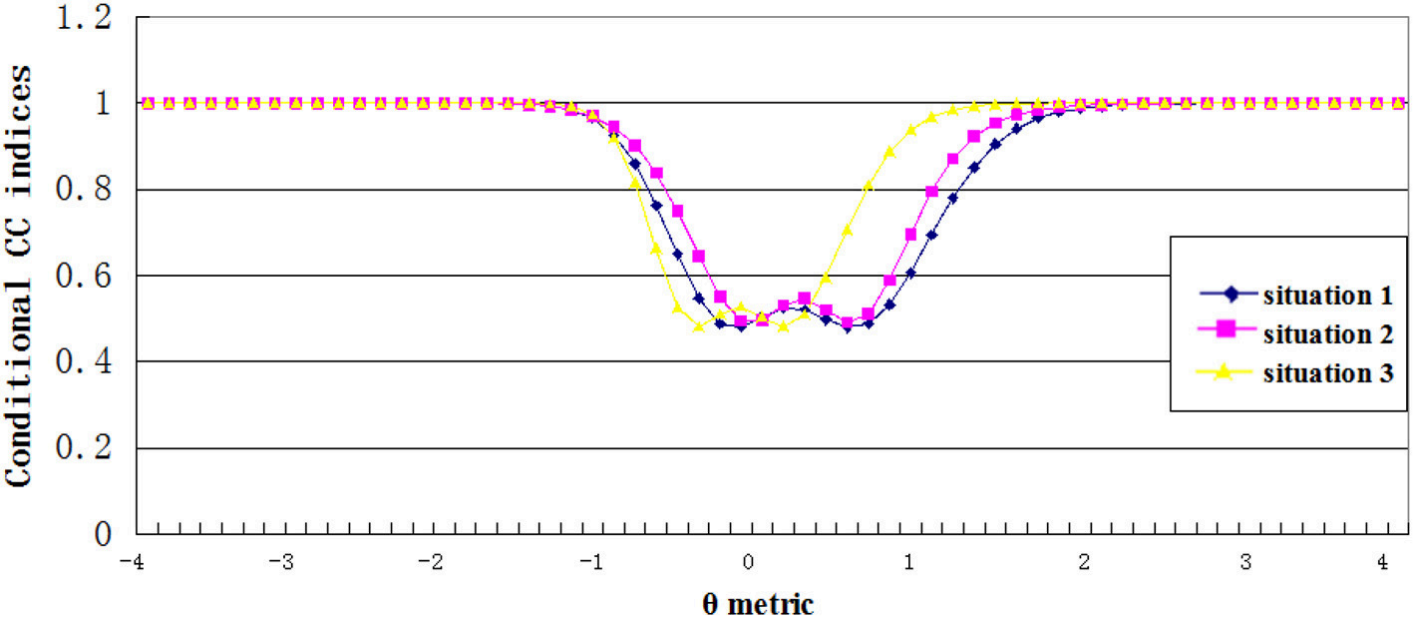
Conditional CC indices for one to one transformation scale.

**Figure 6:**
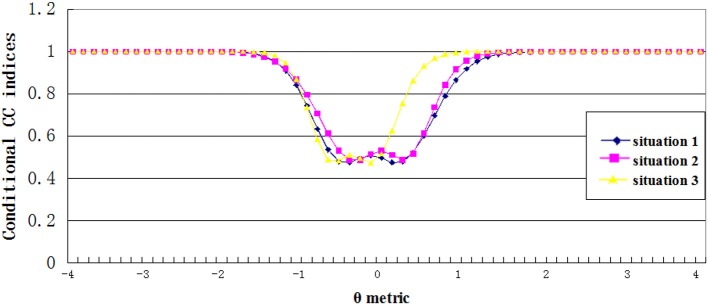
Conditional CC indices for many to one transformation scale.

Figures 5, 6 showed the estimated conditional classification consistency indices (Φ_θ_) at various θ locations with one-to-one and many-to-one score scale. The x-axis is the examinee ability, and the y-axis is the value of the classification consistency for the corresponding ability value. Situations 1, 2, and 3 represent the normal distribution, the positively skewed distribution, and the negatively skewed distribution, respectively.

Three findings can be found from the figures. First, the lowest value of the conditional CC indices appeared when the corresponding ability value was close to the cut scores. This indicates that when an examinee's ability value is close to the cut score, it is hard to classify him or her into the correct category. Second, compared to other two distributions, the negatively skewed distribution has smaller cut score values on the theta scale. This leads to that students with abilities lower than the cut score A in the negatively skewed distribution had smaller CC indices compared to students with same abilities in other two ability distributions, and students with abilities higher than cut score B had larger CC indices compared to students with same abilities in other two ability distributions. Finally, compared to the one-to-one transformation rule, the many-to-one transformation led to smaller cut score values on the theta scale, and therefore, the conditional CC indices curve got a shifting to the left side of the theta scale.

## Empirical study

### Data source

In the empirical study, data from two math achievement tests of a Chinese large-scale assessment were used. Each test was administered to a large group of grade 4 or grade 7 examinees across the country, and the process matrix-sampling design (Lord, 1962) was used to obtain the data. The sample sizes of grade 4 and grade 7 students were 6,863 and 7,144, respectively. The Cronbach's reliability for grade 4 and grade 7 tests were 0.79 and 0.85, respectively. Further details about the two tests can be seen in Table 3. To make transformations from raw score to scale score, one-to-one rule and many-to-one rule were used to get the score scales, which were very similar to the rules in the simulation study. Each score scale has the points ranging from 0 to 100. Sixty and eighty were set at the score scale as cut scores. The values of cut score at the score scale metric and corresponded θ metric can be seen in Table 4.

**Table 3:** Instrument information for real data.

	**Item number**	**Dichotomous items**	**Polytomous items**	**Total score value**	***M***	***SD***
Grade 4 test	24	23 (0,1)	1 (0, 1, 2, 3, 4, 5)	28	19.3	4.74
Grade 7 test	26	25 (0,1)	1 (0, 1, 2, 3, 4, 5)	30	23.1	5.31

**Table 4:** Cut scores at different metric.

	**Cut score values at scale score**		**Corresponded** θ **(one to one)**		**Corresponded** θ **(many to one)**	
	**A**	**B**	**A**	**B**	**A**	**B**
Grade 4 test	60	80	−0.65	0.68	−0.61	0.88
Grade 7 test	60	80	−1.03	−0.13	−1.15	−0.30

Mplus 6.1 (Muthén and Muthén, 2006) was used in the analysis of the real data to check the unidimensionality assumption and the relative degree of fit between the mixed IRT models and the real data. 2PL and GRM were selected as the IRT model combination. PARSCALE and IRT-CLASS were carried out to calibrate the item parameters and compute all CC indices.

### Results

#### Unidimensionality and model fit

Confirmatory factor analysis (CFA) was used to check the unidimensionality. Results (see Table 5) showed both CFI and TLI values were higher than 0.9, which means the selected model combination fit the data very well. The RMSEA was lower than 0.06. Therefore, the error of measurement was acceptable (Hu and Bentler, 1999). The results of CFA confirmed the assumption of unidimensionality.

**Table 5:** CFA for grade 4 and grade 7 math tests.

	**Chi-square**	***df***	***p***	**CFI**	**TLI**	**RMSEA**
Grade 4 test	446.532	252	0	0.945	0.940	0.025
Grade 7 test	869.412	299	0	0.943	0.938	0.032

#### Ability distribution

Table 6 displayed the estimated ability scores computed by PARSCALE. The analysis of the θ score suggested a statistically significant (*p* < 0.05) departure from the normal distribution for both the grade 4 and grade 7 examinees. In actuality, they were subject to the negatively skewed distribution, and the skewness of grade 7 test was even larger than that of the grade 4. Therefore, the data distribution in empirical study was similar to the negatively skewed situation in the simulation study. In addition, the values of Kurtosis were lower than that for normal distribution. It can be inferred that the curve of students' ability distribution might be flatter.

**Table 6:** Descriptive statistics of grade 4 and grade 7 math tests.

	***N***	***Min***	***Max***	***M***	***SD***	***Sk(S_x_)***	***Ku(S_x_)***
Grade 4 test	1,214	−3.010	2.280	0.000	1.000	−0.186(0.070)	−0.458(0.140)
Grade 7 test	1,836	−2.950	1.860	0.000	1.000	−0.312(0.057)	−0.479(0.114)

#### Marginal CC indices

The estimates of the marginal CC indices were summarized in Table 7. The grade 7 data had larger CC indices than grade 4. The increasing number of cut score resulted in the decreasing value of CC indices. When using one cut score, the CC indices with cut score A were higher than with cut score B. The score scale can also make a difference. The many-to-one transformation rule can slightly increase the CC indices comparing to the one-to-one transformation rule when using two cut scores simultaneously.

**Table 7:** Marginal classification consistency indices of grade 4 and grade 7 math tests.

		Φ **indices**			***K*** **indices**		
		**A/B**	**A**	**B**	**A/B**	**A**	**B**
Grade 4 Test	One to one	0.702	0.872	0.827	0.534	0.665	0.571
	Many to one	0.705	0.888	0.815	0.541	0.659	0.605
Grade 7 Test	One to one	0.797	0.939	0.856	0.635	0.755	0.701
	Many to one	0.845	0.951	0.893	0.651	0.736	0.737

#### Conditional CC indices

**Figure 7:**
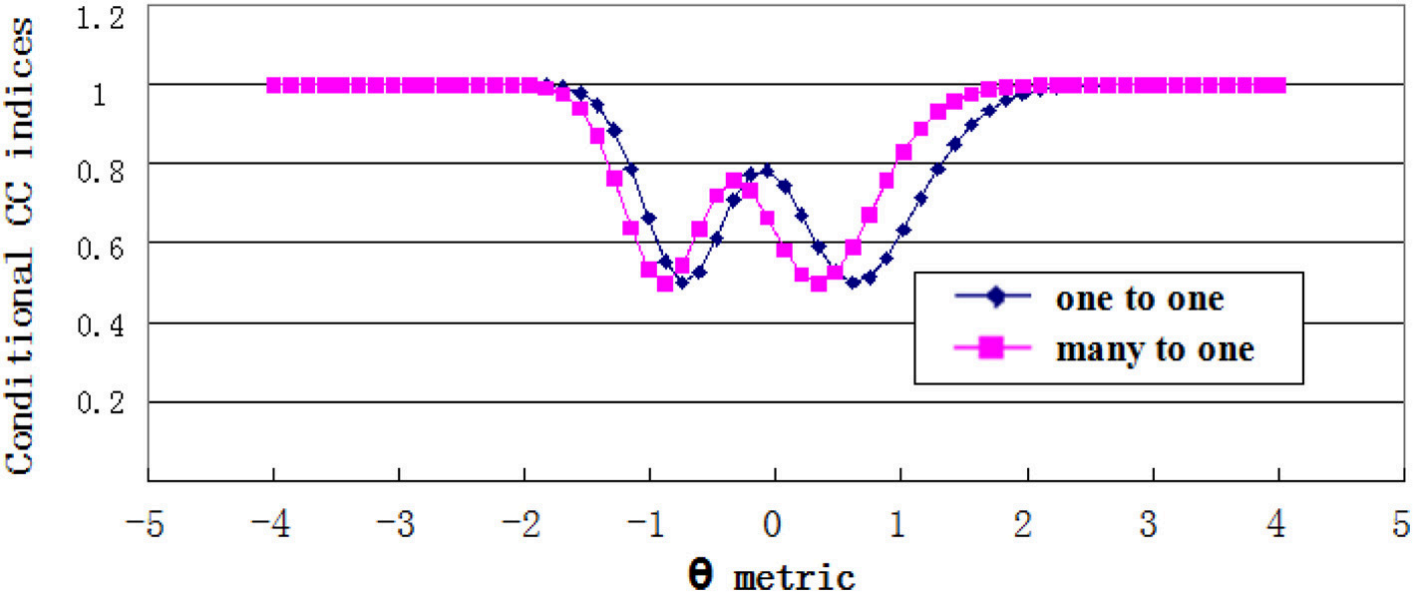
Conditional CC indices for Grade 4 test.

**Figure 8:**
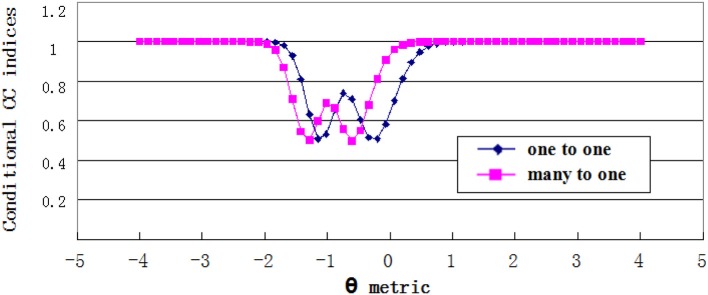
Conditional CC indices for Grade 7 test.

Estimates of conditional CC indices were plotted in Figures 7, 8. For the grade 4 test, the minimum Φ_θ_ had the corresponding θ values of −0.746 and 0.610 on the one-to-one score scale, and −0.881 and 0.339 on the many-to-one score scale. While for the grade 7 test, the θ values were −1.153 and −0.203 on the one-to-one score scale, and −1.288 and −0.6102 on the many-to-one scale. The Φ_θ_ curve showed a wavy pattern with the minimum point having the θ values near the cut scores. This was consistent with the findings in previous studies (e.g., Lee, 2010). The curve of conditional CC indices with many-to-one transformation shifted to the left side from the one-to-one transformation curve, which confirms the results of the simulation study.

## Discussion and limitations

Considered to be one of the most important indicators of test qualities, classification consistency categorizes examinees by comparing their tests scores with given cut scores. The results of this study are helpful for making classification decisions with different ability distributions, cut score numbers and locations, and score scales in the design stage of an assessment, as well as interpreting test performance results to students, parents, and the public. A simulation study and a real data set analysis are conducted to evaluate the CC indices based on Lee's IRT approach.

In this study, Φ and *K* are used as indices of classification consistency decisions. *K*index measures the classification consistency which excludes the probability of chance. However, the performance of *K* index is not as stable as Φ index, and violations exist for *K* index during various conditions. Some researchers note that *K* index needs the assumption of exact marginal proportion and cannot be seen as the reflection of absolute classification consistency (Jeroen, 2007). Other researchers have shown that Φ is more useful for tests using an absolute cut score, whereas *K* is more appropriate when the cut score is determined by the passing/failing proportion (Deng, 2011). In this study, the cut scores were set with absolute values and the results prefer Φ as the classification consistency index.

The negatively skewed distribution contributes to larger marginal CC indices compared to the normal and positively skewed distributions. It can be concluded that for the negatively skewed distribution, the majority of students have higher abilities and higher probabilities of obtaining consistent classifications on parallel forms of test. For the positively skewed distribution, the mass of the distribution is located on the left of the ability scale and the test must be difficult for these examinees, which results in a lower probability of classification consistency and a volatile trend of the standard deviation. Similar to the results of the simulation study, in the real data analysis, the skewness is larger with the grade 7 test than with the grade 4 test, and the marginal CC indices of grade 7 are also larger than those of grade 4.

An interaction occurs between the cut score locations and the ability distributions. The data supports the conclusion that when the ability values of most examinees are around the cut scores, the CC indices will be smaller than when the ability values of most examinees are far from the cut scores. In the real data analysis, the values of CC indices also follow this trend.

Both one-to-one transformation rule and many-to-one transformation rule can get stable CC indices, which can be seen from the similar standard deviations. Since the many-to-one transformation rule alters the distributional shape of the original raw score, the corresponded cut score at θ metric changes, resulting in the varying values of CC indices. The curve of the conditional CC indices with the many-to-one transformation rule tends to shift to the left side of the scale for all ability distributions. The conditional CC indices are underestimated for examinees with lower ability and overestimated for examinees with higher ability. It is suggested that the influence of the transformation must be considered when using the scale score in practice. The present study demonstrates both the one-to-one and the many-to-one transformation rule are acceptable to make transformations. When taking the error of measurement into consideration, the many-to-one transformation scale should being treated with caution.

In this study, the IRT procedure is illustrated using a simulation study and two real data sets consisting of both dichotomous and polytomous item types. The tests prove conclusively Lee's IRT method's validity. Learning the effects of different factors is helpful to facilitate the application of the indices. A limitation of this study is that only 2PLM and GRM are used as model combinations to generate the item response matrix. The influence of the skewed distribution with differing degrees of skewness could also be investigated. In the real data analysis, the kurtosis is not controlled, so it may violate the estimation of the CC indices. Moreover, there are many other factors affecting the CC indices. For example, students' abilities are evaluated based on results of achievement tests. The factors affecting the examinee response process of the achievement test also have effects on the consistency of test (Leighton, 2013). Further research should be done to compare different IRT model combinations with more investigated factors.

In addition, although previous studies regarding the CC indices often considered unidimensional latent ability, it is necessary to consider the multidimensionality in the practical data. According to Lee (2010), if the test is believed to be truly multidimensional, then a multidimensional IRT model could be used to estimate the CC indices under Lee method. Some studies have already considered the estimation of CC indices for multidimensional tests (e.g., Yao, 2013; LaFond, 2014; Wang et al., 2016) and BMIRT software (Yao, 2003) can be used to estimate the multidimensional CC indices. Future research is encouraged to further investigate the performance of the CC indices for multidimensional latent ability.

For the substantive readers, some recommendations are given according to the findings in this study. First, Lee's IRT procedure to estimate the CC indices is recommended to the practitioners because of its good performance which is demonstrated in the simulation and empirical studies of this research. Second, the practitioners should consider the effects of examinee's ability distributions, cut scores, and score transformation rules on the performance of the CC indices. Specifically, too many cut scores should be used with caution as more cut scores lead to lower classification consistency; if most examinees have ability values concentrating on a certain interval of the ability scale, then the cut scores should not be specified on the interval, otherwise it is less possible that the students can be correctly categorized; for the students with ability values around the cut scores, their classification decisions should be reconsidered with caution.

## Author contributions

SZ and TX contributed to the conceptualization and design of the work. JD, PC, SZ, and FC contributed to the analysis and interpretation of data. SZ, JD, and TX were involved in drafting and revising the manuscript. All authors approve the final manuscript submitted.

### Conflict of interest statement

The authors declare that the research was conducted in the absence of any commercial or financial relationships that could be construed as a potential conflict of interest.

## References

[B1] Adam E. W. Hao S. (2012). An evaluation of item response theory classification accuracy and consistency indices. Appl. Psychol. Meas. 36, 602–624. 10.1177/0146621612451522

[B2] Almehrizi R. S. (2013). Coefficient alpha and reliability of scale scores. Appl. Psychol. Meas. 37, 438–459. 10.1177/0146621613484983

[B3] American Education Research Association, American Psychology Association, and National Council on Measurement in Education (2014). Standards for Educational and Psychology Testing. Washington, DC: American Educational Research Association.

[B4] Birnbaum A. (1968). Some latent trait models and their use in inferring an examinee's ability in Statistical Theories of Mental Test Scores, eds F. M. Lord and M. R. Novick (Reading: Addison-Wesley), 397–472.

[B5] Brennan R. L. (2004). A Bootstrap Procedure for Estimating Decision Consistency for Single-Administration Complex Assessments (CASMA Research Report No.17). Iowa City, IA: Center for advanced Studies in Measurement and Assessment, The University of Iowa.

[B6] Cohen J. (1960). A coefficient of agreement for nominal scales. Educ. Psychol. Meas. 20, 37–46. 10.1177/001316446002000104

[B7] Cui Y. Gierl M. J. (2012). Estimating classification consistency and accuracy for cognitive diagnostic assessment. J. Educ. Meas. 49, 19–38. 10.1111/j.1745-3984.2011.00158.x

[B8] Deng N. (2011). Evaluating IRT- and CTT-Based Methods of Estimating Classification Consistency and Accuracy Indices from Single Administrations. Unpublished doctoral dissertation, University of Massachusetts, Amherst, MA.

[B9] Ercikan K. Julian M. (2002). Classification accuracy of assigning student performance to proficiency levels: guidelines for assessment design. Appl. Meas. Educ. 15, 269–294. 10.1207/S15324818AME1503_3

[B10] Hanson B. A. Brennan R. L. (1990). An investigation of classification consistency indexes estimated under alternative strong true score models. J. Educ. Meas. 27, 345–359. 10.1111/j.1745-3984.1990.tb00753.x

[B11] Hendrawan I. Wibowo A. (2011). The Connecticut Mastery Test. Technical Report, Measurement Incorporated, Hartford, CT.

[B12] Hu L. T. Bentler P. M. (1999). Cutoff criteria for fit indices in covariance structure analysis: conventional criteria versus new alternatives. Struct. Equat. Model. 6, 1–55. 10.1080/10705519909540118

[B13] Huynh H. (1976). On the reliability of decisions in domain-referenced testing. J. Educ. Meas. 13, 253–264. 10.1111/j.1745-3984.1976.tb00016.x

[B14] Jeroen D. M. (2007). Agreement and Kappa-type indices. Am. Stat. 61, 148–153. 10.1198/000313007X192392

[B15] Kolen M. J. Hanson B. A. Brennan R. L. (1992). Conditional standard errors of measurement for scale scores. J. Educ. Meas. 29, 285–307. 10.1111/j.1745-3984.1992.tb00378.x

[B16] Kolen M. J. Lee W. (2011). Psychometric properties of raw and scaled scores on mixed-format tests. Educ. Meas. 30, 15–24. 10.1111/j.1745-3992.2011.00201.x

[B17] LaFond L. J. (2014). Decision Consistency and Accuracy Indices for the Bifactor and Testlet Response Theory Models. Unpublished doctorial dissertation, University of Iowa.

[B18] Lathrop Q. N. Cheng Y. (2014). A nonparametric approach to estimate classification accuracy and consistency. J. Educ. Meas. 51, 318–334. 10.1111/jedm.12048

[B19] Lee W. (2010). Classification consistency and accuracy for complex assessments using item response theory. J. Educ. Meas. 47, 1–17. 10.1111/j.1745-3984.2009.00096.x

[B20] Lee W. Brennan R. L. Hanson B. A. (2000). Procedures for Computing Classification Consistency and Accuracy Indices with Multiple Categories. ACT Research Report Series 2000-10. Iowa City, IA: ACT.

[B21] Lee W. Hanson B. A. Brennan R. L. (2002). Estimating consistency and accuracy indices for multiple classifications. Appl. Psychol. Meas. 26, 412–432. 10.1177/014662102237797

[B22] Lee W. Kolen M. J. (2008). IRT-CLASS: A Computer Program for Item Response Theory Classification Consistency and Accuracy (Version 2.0) [Computer Software]. Iowa City, IA: Center for Advanced Studies in Measurement and Assessment, University of Iowa Available online at: http://www.Education.Uiowa.Edu/casma

[B23] Leighton J. P. (2013). Item difficulty and interviewer knowledge effects on the accuracy and consistency of examinee response processes in verbal reports. Appl. Meas. Educ. 26, 136–157 10.1080/08957347.2013.765435

[B24] Li S. (2006). Evaluating the Consistency and Accuracy of Proficiency Classifications Using Item Response Theory. Unpublished doctoral dissertation, University of Massachusetts, Amherst, MA.

[B25] Livingston S. A. Lewis C. (1995). Estimating the consistency and accuracy of classification based on test scores. J. Educ. Meas. 32, 179–197. 10.1111/j.1745-3984.1995.tb00462.x

[B26] Lord F. M. (1962). Estimating norms by item-sampling. Educ. Psychol. Meas. 22, 250–267. 10.1177/001316446202200202

[B27] Muraki E. Bock R. D. (1997). PARSCALE: IRT Item Analysis and Test Scoring for Rating-Scale Data. Chicago, IL: Scientific Software International.

[B28] Muthén L. K. Muthén B. O. (2006). Mplus User's Guide, 4th Edn. Los Angeles, CA: Muthén and Muthén.

[B29] Rudner L. M. (2005). Expected Classification Accuracy. Practical Assessment Research and Evaluation. Available online at: http://pareonline.net/pdf/v10n13.pdf

[B30] Samejima F. (1969). Estimation of latent ability using a response pattern of graded scores in Psychometrika Monograph, Vol. 17 (Richmond, VA: Psychometric Society).

[B31] Subkoviak M. J. (1976). Estimating reliability from a single administration of a criterion-referenced test. J. Educ. Meas. 13, 265–276. 10.1111/j.1745-3984.1976.tb00017.x

[B32] Tong Y. Kolen M. J. (2010). Scaling: an ITEMS module. Educ. Meas. 29, 39–48. 10.1111/j.1745-3992.2010.00192.x

[B33] Wan L. Brennan R. L. Lee W. C. (2007). Estimating Classification Consistency for Complex Assessments (CASMA Research Report No. 22). Iowa City, IA: Center for Advanced Studies in Measurement and Assessment, The University of Iowa.

[B34] Wang T. Y. Kolen M. J. Harris D. J. (2000). Psychometric properties of scale scores and performance levels for performance assessments using polychromous IRT. J. Educ. Meas. 37, 141–162. 10.1111/j.1745-3984.2000.tb01080.x

[B35] Wang W. Song L. Ding S. Meng Y. (2016). Estimating classification accuracy and consistency indices for multidimensional latent ability in Quantitative Psychology Research, eds L. A. van der Ark, D. M. Bolt, W. Wang, J. A. Douglas, and M. Wiberg (Cham: Springer International Publishing AG), 89–103. 10.1007/978-3-319-38759-8_8

[B36] Wheadon C. Stockford I. (2010). Classification Accuracy and Consistency in GCSE and A Level Examinations Offered by the Assessment and Qualifications Alliance (AQA). Manchester: AQA Centre for Education Research and Policy.

[B37] Yao L. (2003). BMIRT: Bayesian Multivariate Item Response Theory [Computer Software]. Monterey, CA: CTB/McGraw-Hill.

[B38] Yao L. (2013). Classification Accuracy and Consistency Indices for Summed Scores Enhanced Using MIRT for Test of Mixed Item Types. Available online at: http://www.bmirt.com/8220.html (Accessed March 1, 2015).

[B39] Yoo H. Sukin M. T. Hambleton R. K. (2009). Evaluating Consistency and Accuracy of Proficiency Classifications Using a Single Administration IRT Method (Final Report). University of Massachusetts, Center for Educational Assessment Amherst, MA.

[B40] Zhang J. Xin T. (2013). Measurement of internet addiction: an item response analysis approach. Cyberpsychol. Behav. Soc. Netw. 16, 464–468. 10.1089/cyber.2012.0525 23505969

